# Object manipulation scaffolds the development of tool use in children

**DOI:** 10.1016/j.isci.2026.117302

**Published:** 2026-08-24

**Authors:** Ellen Soeters, Jake A. Funkhouser, Erik P. Willems, Crickette Sanz, Kathelijne Koops

**Affiliations:** 1 Department of Evolutionary Anthropology, University of Zurich, Zurich, Switzerland; 2 Department of Anthropology, Washington University in St. Louis, St. Louis, MO, USA; 3 Wildlife Conservation Society, Congo Program, Brazzaville, Republic of the Congo; 4 Lester E. Fisher Center for the Study and Conservation of Apes, Lincoln Park Zoo, Chicago, IL, USA

**Keywords:** object manipulation, tool use, social learning, environmental opportunities

## Abstract

Our species’ extensive reliance on tool use is a key aspect of what makes us human, yet naturalistic data on the social and environmental drivers of tool-use development in children remain scarce. We examined the development of tool use in 31 children in Swiss daycares (age: 0.5–4.5 years, 17 females, *n* = 467 video focal follows). All children frequently engaged with objects. Simple object-manipulation bouts became shorter with age, and the number of complex tool-use bouts increased. Social learning opportunities increased the frequency of material engagement, whereas the mere presence of others reduced it. Higher object availability predicted both higher frequency and complexity of material engagement. In sum, children were intrinsically motivated to manipulate objects, and environmental and social factors further influenced tool-use development. Our naturalistic study paves the way for comparisons with non-human species—crucial to understand the evolution of material culture and cognition.

## Highlights

• Naturalistic observations provide support for preparation for tool-use hypothesis
• With age, object manipulation decreases, while tool use increases
• Social learning opportunities drive frequency of material engagement
• Object availability predicts both frequency and complexity of object engagement

## Graphical abstract

**Figure:**
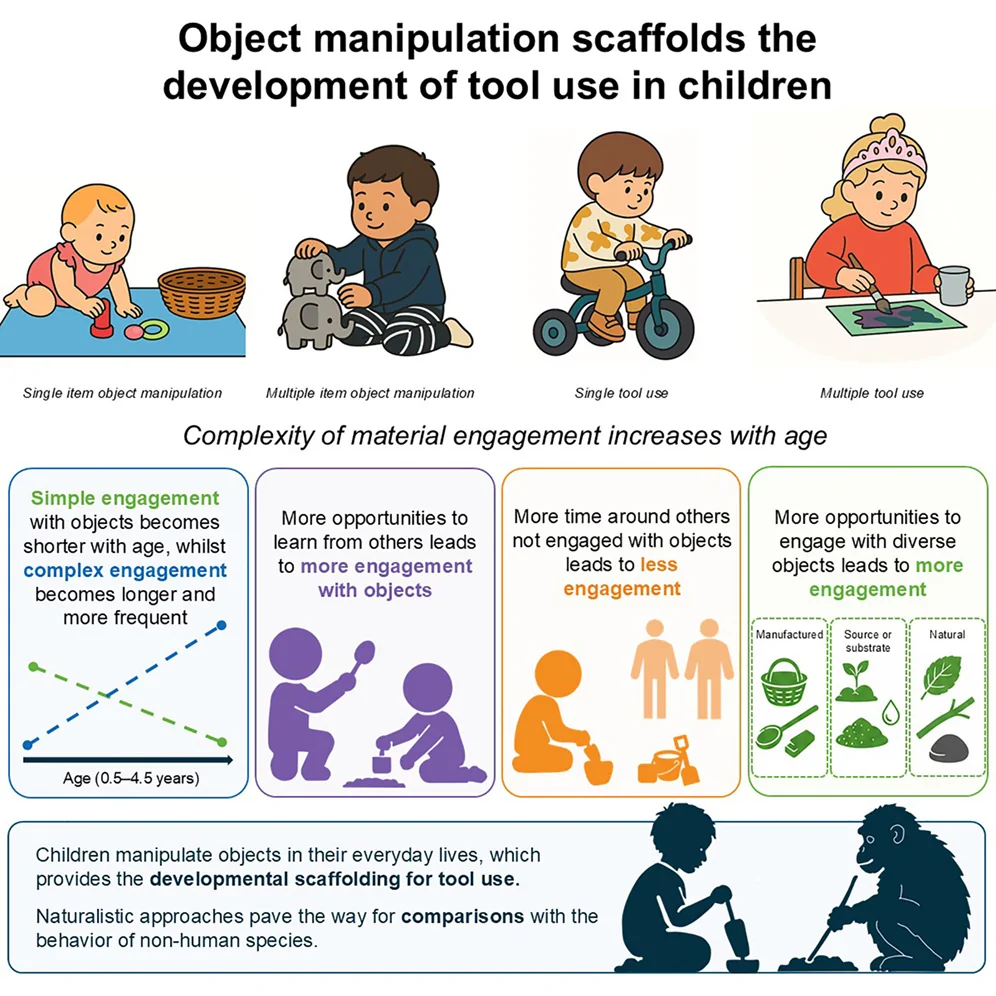

## Teaser

Natural sciences; Biological sciences; Neuroscience; Clinical neuroscience

## Introduction

Human uniqueness is in part defined by our complex and extensive use of technology, yet little is known about the ontogeny and drivers of children’s tool-use skills in naturalistic settings.^1^ Previous research in human and non-human primates has provided valuable insights into the foundations of tool-related skills, and proposed, but rarely tested, is the hypothesis that object manipulation ontogenetically scaffolds tool use (i.e., preparation for tool-use hypothesis^2^^,^^3^^,^^4^^,^^5^). We use the term “scaffolds” here to refer to object manipulation appearing earlier in development and being associated with the later emergence of tool use, rather than implying a direct causal mechanism (cf. Kahrs and Lockman^4^, and Lockman^6^). Object manipulation by young individuals, such as object play and exploration, provides a plethora of opportunities to develop the physical motor skills and the causal understanding necessary to successfully use tools later in development.^4^^,^^5^^,^^7^^,^^8^^,^^9^^,^^10^ Hence, learning via object manipulation may act as a cognitive stepping-stone for the development of more complex tool use,^5^^,^^11^ a pattern also observed in other species. For instance, early percussive object manipulation precedes nut cracking in capuchin monkeys,^12^ and combinatory object manipulation during play is associated with functional tool use in New Caledonian crows and Goffin cockatoos.^13^ However, most studies in human children to date have focused solely on tool use in experimental tasks.^5^^,^^14^^,^^15^ Thus, how everyday developmental environments shape the emergence of tool-use skills in children remains understudied,^16^ and the preparation for tool-use hypothesis warrants explicit investigation. We address this gap by examining the ontogenetic transition between object manipulation and tool use in young children, along with the social and environmental drivers of these behaviors, which are needed to understand the developmental factors that shape the emergence of tool use.

**Figure 1:**
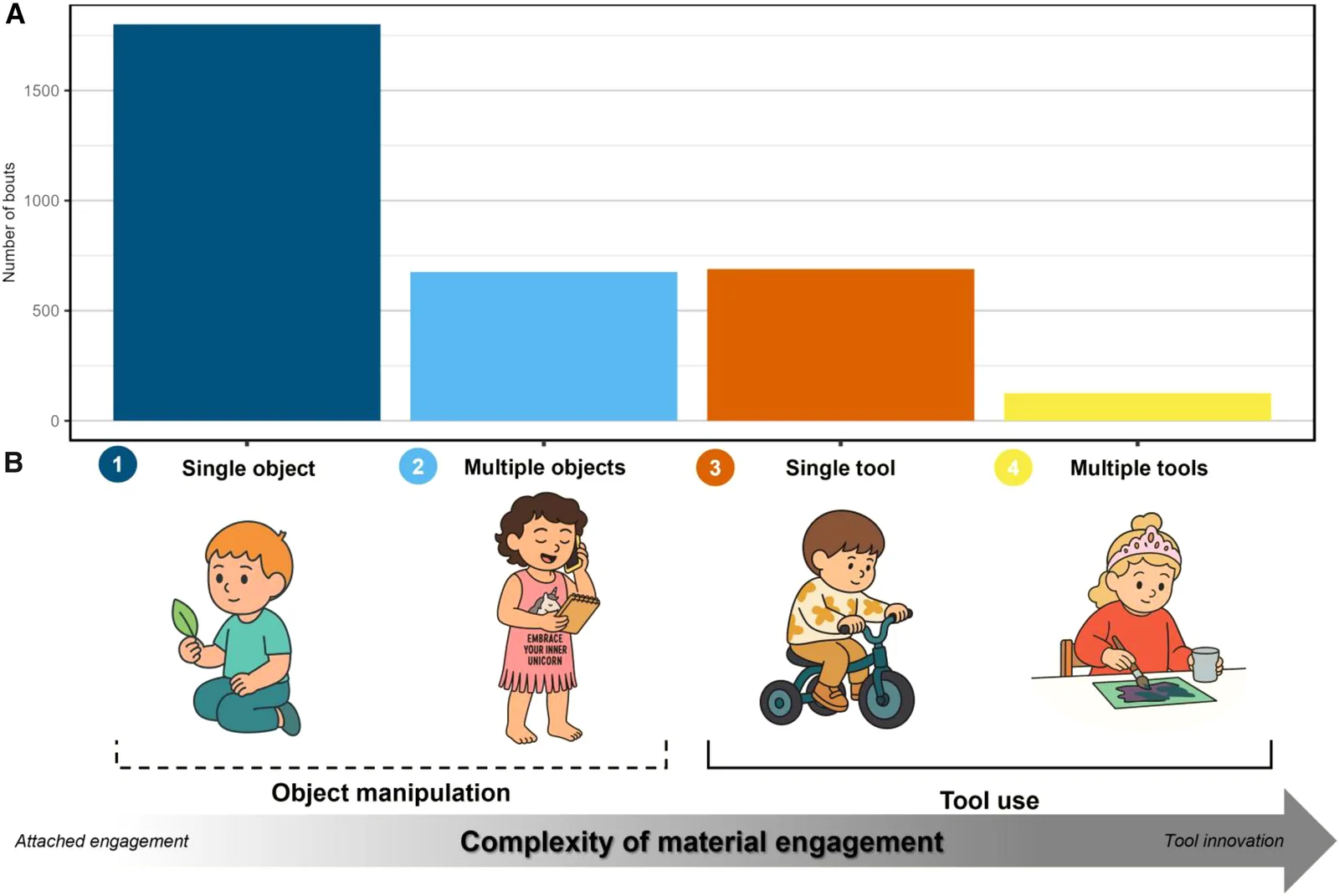
Scale of material engagement (A) Observed number of bouts per complexity level of material engagement. (B) Scale of material engagement, from attached engagement to detached object manipulation, tool use, and tool innovation.

Human children are commonly described to develop their tool skills between 2 and 6 years of age, though infants often engage with objects as early as 6 months of age (reviewed in Koops and Sanz^1^) and show initial tool use by 8–24 months of age in experimental contexts.^17^^,^^18^ Object manipulation—the continuous engagement of object(s) with the hands, feet, or mouth^11^—can vary in the level of complexity, namely, the number of objects involved.^19^ Object manipulation differs from tool use—the external employment of a single detached or manipulatable attached environmental object to alter more efficiently the form, position, or condition of another object, organism, or the user themselves^20^—which can vary in complexity, from engaging with a single tool to using multiple tools. We henceforth use the umbrella term “material engagement” to refer to this scale of detached object- and tool-directed behaviors—encompassing simple object manipulation and more complex tool use—representing a continuum of increasing complexity (Figure 1B). Several studies indicate that infants below 2 years of age engage in such simple object manipulation for >50% of their observed free play time, both at home^21^^,^^22^^,^^23^ and in daycare settings.^24^ As infants become more familiar with objects and increase their tool-use proficiency, they have been reported to engage in object manipulation for shorter durations.^25^

Hence, the prevalence and type of material engagement change as children age. For example, object exploration peaks in infancy and then begins to decline as children become more familiar with the functions and properties of objects.^26^^,^^27^ Furthermore, simple types of object manipulation (e.g., touching and carrying) gradually develop into more goal-directed, functional, and complex manipulations. Therefore, it has been suggested that this increase in complexity is associated with cognitive and brain development,^22^^,^^28^ particularly the maturation of the executive and sensorimotor systems involved in motor planning, working memory, and hierarchical action sequencing.^4^^,^^29^ As children develop their cognitive skills, they progress from combining and stacking (12–24 months^27^^,^^30^), to engaging in simple tool use like raking and spooning (18–30 months^18^) and eventually to using sets of multiple tools (4–8 years^31^^,^^32^).

Children engage with a diverse range of objects in their daily lives.^15^^,^^16^^,^^22^^,^^24^^,^^33^^,^^34^ Objects can range from natural items (e.g., sticks and leaves), to manufactured toys and tools (e.g., balls, baskets, and utensils).^15^^,^^22^^,^^24^^,^^33^^,^^34^ By ∼12 months of age, the transition to self-locomotion (i.e., crawling and walking^21^) increases children’s access to objects,^22^^,^^35^ leading to more frequent object manipulation.^21^^,^^35^ Social learning—which facilitates the transmission of behaviors and skills within and across generations—likely plays a key role in shaping early material engagement.^36^^,^^37^ Experimental studies have demonstrated that infants are more likely to imitate behaviors that include objects (e.g., shaking objects) than those that do not (e.g., dancing and pointing^38^). Early naturalistic observations in daycares have also reported that most interactions between infants involved objects.^39^ The importance of social learning is evident in children as young as 3–4 years of age who, when working alone, were less successful at a novel tool-use task than those working with social partners.^40^ Together, these findings suggest that social learning opportunities and object availability in the everyday environment of children may shape developmental patterns. However, the roles of these potential drivers have yet to be investigated systematically to assess their influence on the development of tool use in young children.

### The current study

In this study, we investigated the development of tool use and the influence of social and environmental factors on material engagement in young children in Swiss daycares. To date, few studies have explored how children engage with objects outside of an experimental setting,^5^^,^^15^ such as in home or daycare environments.^22^^,^^24^ Natural observations in daycares offer a unique perspective on tool-use development, since children spend much of their time engaging freely with the environment in a social setting.^24^ First, we asked: do developmental trajectories of material engagement show patterns that support the preparation for tool-use hypothesis? If object manipulation is associated with the development of tool-use skills, we predict that as children age, they will shift from simpler object manipulation to more complex tool use. Alternatively, if object manipulation is not associated with preparation for tool use, no age-related shift from object manipulation to tool use will be observed. In addition, we expect that as children develop, they will engage with objects more frequently and use tools for longer durations as they expand their tool repertoire. Second, we asked: how do social and environmental opportunities influence children’s material engagement? If social and environmental opportunities facilitate engagement, we predict that when children are in close proximity to other individuals manipulating objects (i.e., social opportunity) and when materials are more readily available (i.e., environmental opportunity), they will engage with objects more frequently and for longer durations. Furthermore, we predict that children’s engagement with objects will differ between settings. When children are inside, typically in closer proximity to others, we predict that they will engage with objects more frequently. When children are outside, we expect them to engage in more complex material engagement due to a greater availability of diverse material types, specifically manufactured toys/tools, natural objects, and source material. By examining children’s material engagement in naturalistic settings, an approach that has been largely overlooked, this study investigated whether age-related patterns are consistent with object manipulation ontogenetically preceding tool use. By further explicitly testing how social and environmental factors shape this trajectory, we endeavored to take a step toward placing human tool-use ontogeny within a broader comparative framework.

## Results

We observed 31 children (aged 6–58 months, mean = 33 months, SD = 13 months) across five separate groups in two daycares in Zurich, Switzerland. Using focal sampling with continuous video recording,^41^ we collected 467 video focal follows (10 min each) over two combined data collection periods in 2023 and 2024 (6 months total). Video observations were recorded both indoors (*n* = 131) and outdoors (*n* = 336).

Across 467 video focal follows, we scored a total of 2,163 detached material engagement bouts (per video focal follow: average = 7.7, range = 1–27). A bout was defined as when a participant actively engaged, for a minimum of 3 s, with any detached manipulatable object(s) by manipulating the object with their hands, feet, or mouth.^11^ These bouts accounted for 50.5% of all visible activity (35.6 h). Object manipulation accounted for 75.2% of all material engagement (see Figure 1A). Engagement with a single item accounted for more than half of all material engagement (54.7%) and engaging with multiple items accounted for 20.5%. Tool use accounted for 24.8% of all material engagement and was observed in every child. The youngest child to demonstrate tool use was 6–7 months of age, with the earliest instances comprising self-directed tool use (e.g., bottle use). The use of a single tool was observed 20.9% of the time. Using multiple tools, the most complex level of material engagement measured, accounted for the least amount of time (3.9%). While we did not distinguish between different types of tool use in our models, we observed a range of tool use types across contexts: feeding (e.g., using utensils), self-maintenance (e.g., using tissues), social (e.g., chasing with a stick), object play (e.g., riding a tricycle), and construction (e.g., building sandcastles). Material engagement was quantified and analyzed in three distinct ways. In the first model, we tested the frequency of material engagement bouts for the entire video focal follow, fitting a negative binomial generalized linear mixed model (GLMM) (see Table S1). To explore the duration of material engagement bouts, we fit a Gaussian GLMM (see Table S2). To examine variation in the complexity of material engagement bouts, and test for a shift from simpler object manipulation to more complex tool use, we fit a cumulative link mixed model (CLMM) (see Table S3). For both the duration and complexity of material engagement at the bout level, we used our scale of material engagement (see Figure 1B). All models significantly outperformed their respective null models (likelihood ratio tests, *p* = < 0.001).

### Developmental trajectories

**Figure 2:**
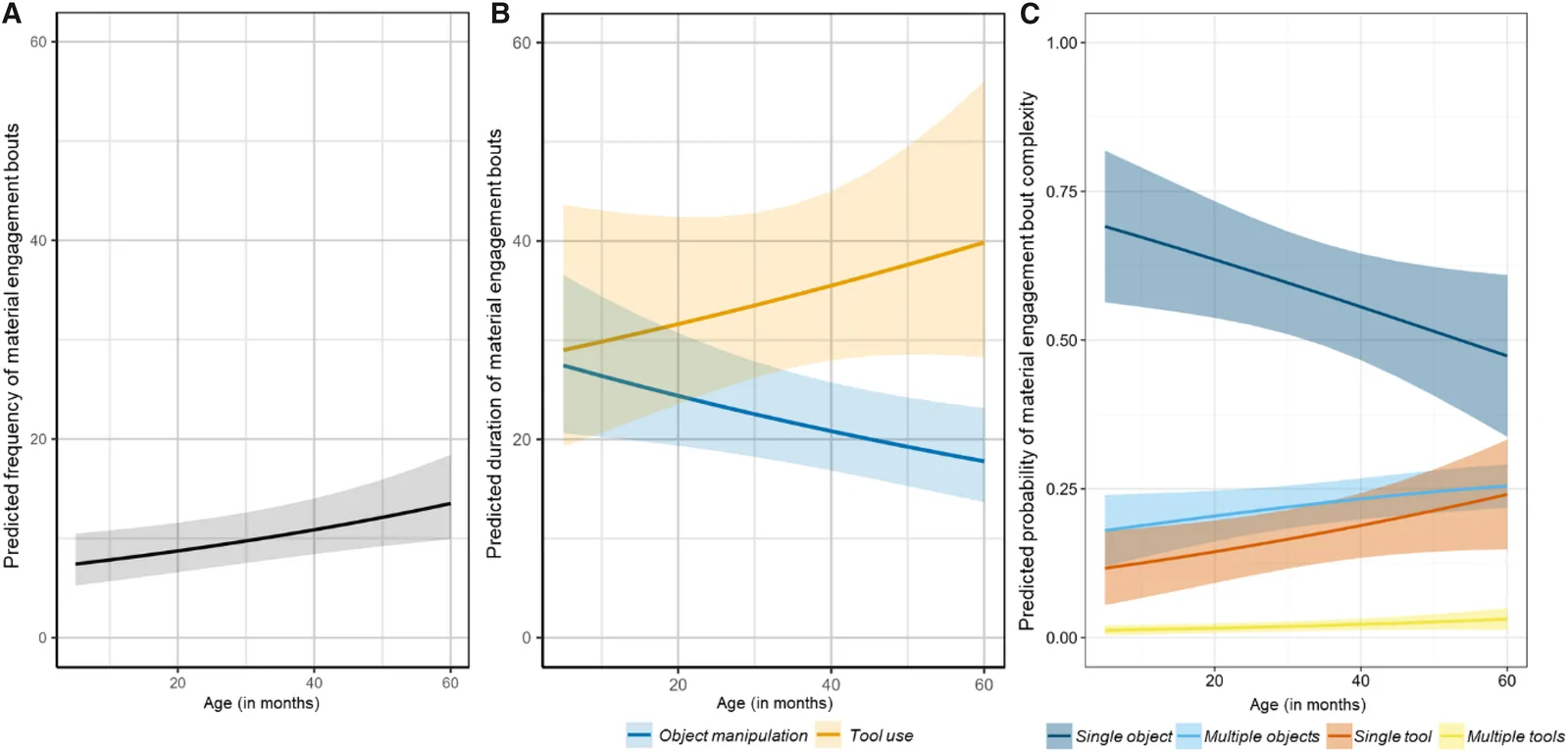
Age predicts material engagement bouts The developmental trajectories of predicted (A) frequency, (B) duration, and (C) complexity of material engagement bouts across age. Continuous covariates are held at their mean values; categorical covariates are fixed at reference levels (context: free play; sex: female; location: indoors). Lines represent model-predicted estimates, and shaded bands indicate 95% confidence intervals.

We first examined how age predicted the frequency, duration, and complexity level of material engagement to test whether developmental patterns were consistent with the preparation for tool-use hypothesis. We found that children engaged with objects more frequently with increasing age (β = 0.01, confidence interval [CI]: [0.01, 0.02], SE = 0.01, *z* = 2.84, *p* = 0.01), indicating that older children engaged with objects more often than younger children (Figure 2A). To examine developmental changes in the duration of material engagement, we tested an interaction between age and engagement type, simplified to a binary scale between simpler object manipulation and more complex tool-use bouts. The duration of object manipulation bouts decreased with age (β = −0.01, CI: [–0.01, 0.01], SE = 0.01, *z* = −2.39, *p* = 0.02), whereas the duration of tool-use bouts increased (β = 0.01, CI: [0.01, 0.02], SE = 0.01, *z* = 2.56, *p* = 0.01; Figure 2B). Therefore, as children get older, their tool-use bouts become longer relative to their object manipulation bouts. We then examined how age and duration predicted the complexity of material engagement bouts. Longer bouts were more likely to involve greater complexity of engagement (β = 0.29, CI: [0.21, 0.38], SE = 0.04, *z* = 6.92, *p* = 0.001). In addition, older children were more likely to engage in more complex material engagement (β = 0.02, CI: [0.01, 0.03], SE = 0.01, *z* = 2.00, *p* = 0.045; Figure 2C). We found no significant sex differences across our three models (see Tables S1–S3).

### Social factors

**Figure 3:**
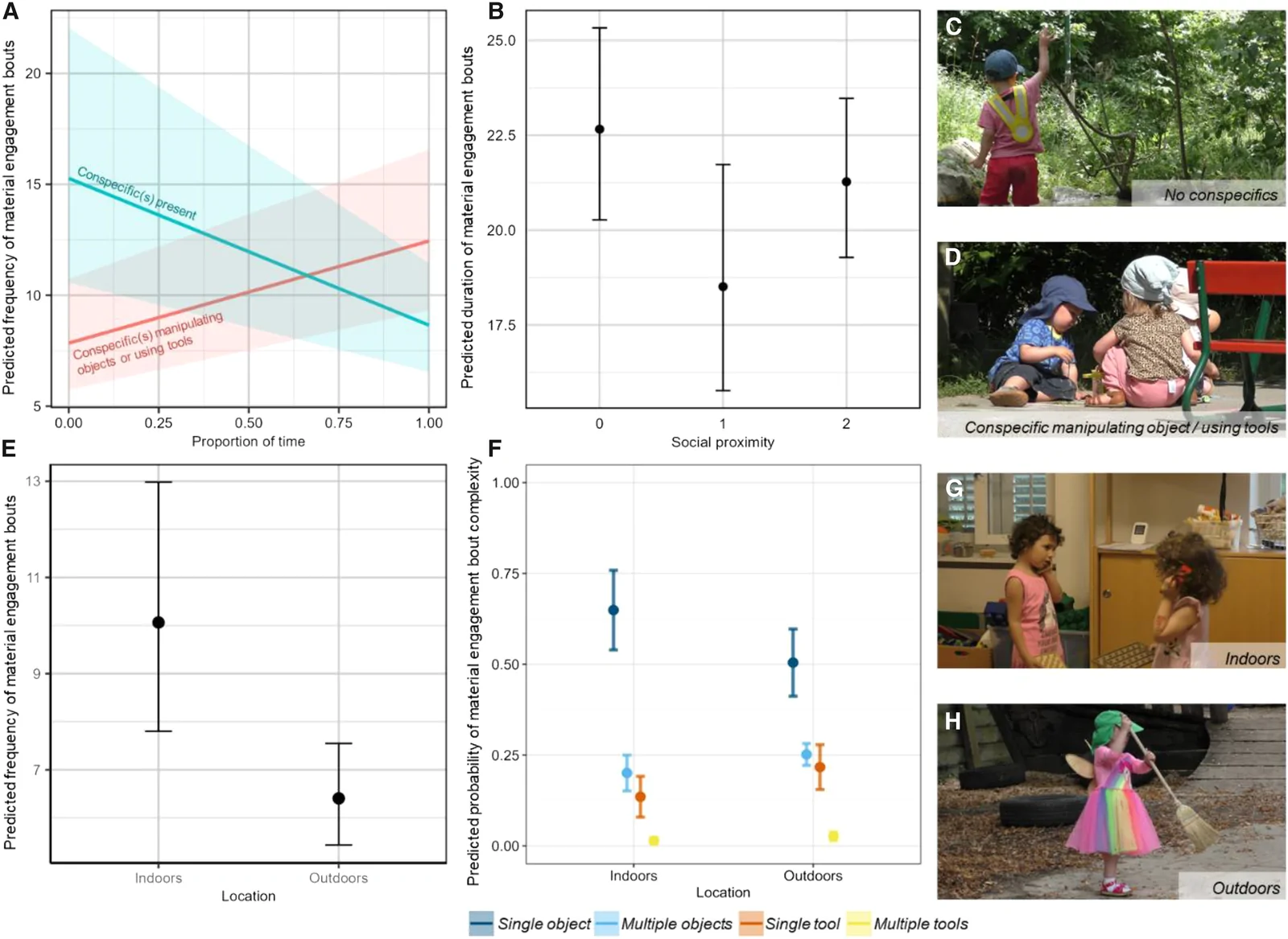
Social factors and location predict material engagement bouts Predicted effects of social proximity and opportunity on (A) the frequency and (B) the duration of material engagement bouts (social factors scored on the *x* axis as 0 = alone, example in C; 1 = social proximity and 2 = social opportunity, example in D); predicted effects of location on (E) the frequency and (F) the ordinal complexity level of material engagement, with image examples of indoor (G) and outdoor (H) settings. Continuous covariates are held at their mean values; categorical covariates are fixed at reference levels (context: free play; sex: female; location for plots A and B: indoors). Lines and points represent model-predicted estimates. Shaded bands and error bars indicate 95% confidence intervals.

We found that social proximity and social opportunity strongly influenced how often children engaged with objects. When children spent more time in close proximity to others (i.e., social proximity) they engaged with materials less frequently overall (*β* = −0.57, CI: [–0.95, −0.19], SE = 0.19, *z* = −2.92, *p* = 0.01). This effect was tested regardless of a nearby individual’s behavior. In contrast, when children spent more time in close proximity to others who were engaging with objects (i.e., social opportunity), focals themselves engaged with objects more frequently (β = 0.46, CI: [0.14, 0.78], SE = 0.16, *z* = 2.85, *p* = 0.01; Figure 3A). Next, we examined how social proximity and opportunity influenced the duration of engagement with objects. We did this by examining three levels: bouts that occurred when children were alone, bouts that occurred in proximity to other individuals engaged in other behaviors (i.e., social proximity), and bouts that occurred in proximity to others engaged with objects (i.e., social opportunity). Manipulation bouts were shorter when children were in proximity to others who were not engaging with objects (β = −0.20, CI: [−0.37, −0.03], SE = 0.09, *z* = −2.37, *p* = 0.02) than when children were alone. In contrast, no difference in duration was found between when children were alone and when children were in proximity to object-specific engagement (Figure 3B). Furthermore, neither social proximity nor opportunity significantly predicted more complex tool-use engagement (see Table S3).

### Environmental factors

The effects of object availability on material engagement differed by material category. The presence of natural items (β = 0.56, CI: [0.30, 0.81], SE = 0.13, *z* = 4.31, *p* = 0.01) and human-made tools (β = 0.49, CI: [0.25, 0.72], SE = 0.12, *z* = 3.99, *p* = 0.01) predicted an increase in the frequency of material engagement, whereas source materials showed no effect (β = 0.14, CI: [−0.10, 0.37], SE = 0.12, *z* = 1.13, *p* = 0.26). Object availability did not significantly affect the duration of object engagement bouts (see Table S2). For engagement complexity, the presence of source material (β = 0.28, CI: [0.02, 0.55], SE = 0.14, *z* = 2.08, *p* = 0.04) and human-made tools (β = 0.36, CI: [0.11, 0.61], SE = 0.13, *z* = 2.82, *p* = 0.01) predicted more complex bouts of material engagement, whereas natural items did not (β = −0.07, CI: [−0.39, 0.25], SE = 0.16, *z* = −0.41, *p* = 0.68).

### Settings and contexts

Children manipulated objects more frequently when they were indoors compared with when they were outdoors (β = −0.45, CI: [−0.75, −0.16], SE = 0.15, *z* = −2.99, *p* = 0.01; Figure 3C), but bouts of material engagement outdoors were more complex (β = 0.61, CI: [0.14, 1.09], SE = 0.24, *z* = 2.52, *p* = 0.01; Figure 3D). The duration of material engagement bouts did not differ between indoor and outdoor settings (β = −0.09, CI: [−0.30, 0.13], SE = 0.11, *z* = −0.79, *p* = 0.43). Furthermore, bouts occurred significantly more frequently in the context of play (β = −0.93, CI: [−1.14, −0.71], *p* = 0.01) as compared with non-play contexts (i.e., structured activities, traveling, feeding). However, play contexts had no effect on bout durations or complexity levels (see Tables S2 and S3).

## Discussion

We used naturalistic observations to investigate the development of tool use in young children and to assess the influence of social and environmental factors. Our findings provide the first empirical support consistent with the preparation for tool-use hypothesis in humans.^2^^,^^3^^,^^5^^,^^11^ We demonstrate that material engagement is central to early ontogeny, with children spending approximately half of their time interacting with objects. Throughout development, children demonstrate increasingly complex material engagement. Our cross-sectional observations show that simpler object manipulation is more predominant at younger ages while more complex tool use is more prevalent in older children, a pattern consistent with the preparation for tool-use hypothesis. Older children spend more time engaging in tool use than younger children, suggesting that object manipulation may provide children with the opportunity to develop the cognitive and motor skills associated with tool use. Moreover, we show that the development of tool use is shaped by social and environmental factors, highlighting both the influence of social opportunity and object availability on children’s material engagement. Specifically, proximity to others who were engaging with objects (i.e., social opportunity) increased the frequency of material engagement, and increased object availability was associated with higher frequency and complexity of material engagement. Overall, our results emphasize the importance of both intrinsic developmental processes and extrinsic influences in shaping the development of object manipulation and, subsequently, tool use.

### Preparation for tool use

We investigated whether developmental patterns were consistent with the preparation for tool-use hypothesis. First, we examined whether material engagement bouts occurred more often, and whether they became longer and more complex with increasing age. As expected, bouts of material engagement occurred more often in older children. Children under 12 months old had an average of 6.5 material engagement bouts per video focal follow (51% of time), whereas children over 4 years of age had an average of 8.7 bouts (58% of time), highlighting that children throughout their development frequently engage with objects. Our findings are consistent with previous observational studies showing that infants and young children across cultural settings frequently interact with objects in their everyday environments.^21^^,^^22^^,^^23^^,^^24^

When we examined individual bouts of material engagement, we found clear developmental differences. Bouts of simple object manipulation were significantly shorter in older children, while bouts of more complex tool use were sustained for longer durations by older children (Figure 2B). Single-object manipulation was the most common type of engagement for the youngest children in our sample. Although this type of engagement was present in children of all ages, it decreased steadily with age as children displayed increasingly more complex material engagement. Our findings are consistent with previous research that reports object exploration peaking in infancy,^26^^,^^27^ potentially helping to establish sensorimotor coordination patterns.^4^ Taken together, these findings are consistent with an ontogenetic shift from exploratory object manipulation toward more sustained and goal-directed tool use. This transition from object manipulation to tool use may reflect development across neural systems. Sensorimotor regions mature relatively early and support basic object manipulation from infancy, whereas the prefrontal cortex—which supports executive functions (e.g., working memory, inhibitory control, action sequencing) necessary for increasingly complex and goal-directed tool use—develops at a slower rate over the first 5 years of life.^29^^,^^42^ Although overall tool use increased with age, there were few observations of the most complex level: multiple tool use (*n* = 96). While this likely reflects how genuinely rare these complex skills are in children aged under 5 years, the developmental patterns of complex tool use warrant cautious interpretation. Future research should investigate whether the use of multiple tools simultaneously continues to increase developmentally, as well as investigate the developmental trajectories of other types of complex tool use in naturalistic settings, such as the use of tool sets and sequential tool use.^20^^,^^32^ Additionally, future longitudinal examinations of exactly how children engage with objects are necessary to fully understand whether early object manipulation prepares the ground for later tool use. Combining a longitudinal approach with child-friendly neural recording methods would allow us to further examine whether this developmental trajectory is associated with corresponding changes in prefrontal and sensorimotor neural activity.

Previous research has demonstrated that tool use develops in children aged between 2 and 6 years (reviewed in Koops and Sanz^1^), with some experimental evidence of tool use emerging in children aged 8 –24 months.^17^^,^^18^ Our findings show that over 15% of all tool use behaviors were observed in children aged under 2 years, with the youngest infants (aged 6–9 months) already demonstrating simple self-directed tool use (e.g., using bottles to drink, both with caregiver assistance and independently). Unlike experimental contexts that involve specific tasks and instructions, we observed a wide range of non-instructed tool-use skills across different contexts, such as drawing with crayons, using tissues to clean bodily fluids, drinking from bottles, using shovels to dig sand, and riding tricycles. Our findings indicate that tool use likely emerges earlier in more natural and unstructured settings where children can engage freely with objects. Cross-cultural studies are essential to evaluate how universal the timing of the transition from object manipulation to tool use is across human cultures.

Our results provide no evidence of sex differences in material engagement (see Tables S1–S3), suggesting that children in our daycare sample display similar developmental trajectories in object manipulation and tool use across sexes. In a previous study of wild chimpanzees in Kalinzu (Uganda), an examination of the preparation for tool-use hypothesis was specifically aimed at testing for developmental sex biases in object manipulation corresponding to sex biases of adult tool-use behaviors.^11^ Similarly, sex differences in humans may be more likely in communities and cultural contexts where adult tool use is strongly gendered (e.g., Moser et al.^43^). However, few studies of human children have utilized natural observations in everyday settings, limiting generalizations of when and how sex differences in tool-use development may arise.^26^

### Social factors

We predicted that social factors would shape children’s material engagement. As expected, the frequency of material engagement increased when children were in close proximity to other individuals who were interacting with objects (i.e., social opportunity) but decreased when children were in close proximity to other individuals engaged in other activities (i.e., social proximity). This pattern suggests that the mere presence of others does not promote material engagement, but instead the rate at which children engage with objects appears to be facilitated by close-by models also engaged in object interaction. Research shows that from as early as 9 months of age, children attend to object-specific actions,^44^ displaying active object-specific copying by 12–18 months of age,^45^^,^^46^ and engage in overimitation by 3–4 years of age.^47^ While we could not explicitly measure whether social learning occurred, our results are consistent with social learning taking place, ranging from stimulus or local enhancement^1^^,^^48^ to higher fidelity transmission, such as emulation and imitation.^47^^,^^49^ Disentangling social learning mechanisms in naturalistic settings requires future studies to not only assess gaze and attention but also to identify from whom children learn.^37^^,^^50^^,^^51^ Furthermore, the observed effect of being in proximity to models, and not of mere proximity, may distinguish human children from other primates, where studies have typically reported increased object exploration in the presence of conspecifics (i.e., individuals of the same species) more generally. For example, immature wild orangutans increase their exploratory behaviors when in association with conspecifics,^52^ and captive chimpanzees show higher rates of object exploration when near conspecifics.^53^ Our findings support the idea that, in children, being close to others promotes object engagement only when conspecifics actively interact with objects, thereby providing potential opportunities for social learning.

The frequency and duration of material engagement bouts decreased when children were in close proximity to others not engaging with objects. Shorter and more frequent bouts may reflect competition for attention in social contexts, particularly in daycare settings where children may need to divide their attention between interacting with objects and observing social interactions. Interestingly, previous research in daycare settings has shown that most social interactions between children are object related,^39^ suggesting that material engagement may play a central role in structuring social interactions. While most research has focused on controlled experimental contexts; naturalistic observations may provide valuable insight into how, and when, children attend to other individuals’ interactions with objects in everyday settings. Future studies could examine how observational learning of object-related skills operates during unstructured play, particularly in environments such as daycares where children spend much of their time interacting with peers.^24^^,^^37^^,^^51^^,^^54^ An investigation of the social learning mechanisms that support children’s material engagement is particularly important to understand the emergence of more complex tool-use skills.

### Environmental factors

Finally, we examined the role of environmental factors on tool-use development. As predicted, the frequency of material engagement increased when children were in close proximity to objects. This finding supports the idea that access to objects provides opportunities for exploration and interaction, allowing children to learn about the physical properties and affordances of materials through direct engagement. In contrast, object availability did not increase the duration of material engagement bouts. Instead, it increased the likelihood that children engaged in tool use, particularly when pre-made tools or source materials were present. Our results indicate that the availability of objects is important for the development of object-related skills and that the availability of tools in the environment is linked to the emergence of tool use behaviors in young children. Similarly, we found that the availability of source materials, such as sand or soil, promotes tool use, indicating that these materials are not simply passive features of children’s environments but actively shape opportunities for learning about tool use.

Differences in engagement between indoor and outdoor settings further highlight the importance of environmental opportunities. Although children spent comparable amounts of time in proximity to objects in both locations, the frequency of engagement was higher indoors, whereas outdoor settings increased the likelihood of tool use. These observations align with research across diverse cultural contexts showing that children frequently use natural materials as tools during outdoor play.^15^^,^^16^^,^^22^^,^^24^^,^^33^^,^^34^ Outdoor environments likely provide an abundance of opportunities for children to manipulate a wider diversity of materials and actively change their environment that is not possible in fixed indoor spaces (e.g., using toys and tools to modify natural substrates such as sand). Our findings highlight that providing developing minds with ample opportunities to engage with various types of objects across settings may play an important role in the emergence of tool-use skills. Beyond the factors we measured here, future work should consider measuring additional environmental and social influences that might affect material engagement in children, such as weather conditions (outdoors), time of day, group size and composition, and caregiver behavior.

### Conclusions

This study provides one of the first empirical tests of the preparation for tool-use hypothesis using naturalistic observations, offering important evidence consistent with object manipulation serving as a precursor to the development of tool use in children. Across early childhood, material engagement shifts from simple exploratory object manipulation to increasingly complex and sustained tool use, with outdoor access to different types of materials associated with this developmental pattern. Moreover, our results emphasize that this developmental trajectory is shaped by social and environmental inputs. Social contexts appear to be selectively facilitative, driving the frequency of material engagement only when conspecifics are themselves engaging with objects and using tools, while the mere presence of others has the opposite effect. The diversity of available materials, including manufactured tools, natural objects, and source-substrate materials, additionally emerges as an important driver of material engagement. By capturing everyday interactions with conspecifics and objects in a natural setting, our study provides a more in-depth understanding of how the unique technological skills of our species develop.

While advancing our understanding of the development of tool use in humans, our study also provides key avenues for future research. Firstly, cross-cultural studies are essential to test whether the developmental trajectories described here are universal or whether culturally specific learning acts as an additional influence on material engagement. Such insights would help us to further disentangle what drives the emergence of cumulative tool use in children older than we studied here. Secondly, our naturalistic methods pave the way for direct comparisons with other species, in which object manipulation has similarly been shown to scaffold the later emergence of tool use.^12^^,^^13^ Such interspecific comparisons are essential for situating human tool use ontogeny within a broader evolutionary framework to identify the foundation of our species’ material culture. Ultimately, our findings are consistent with the development of tool use in young children not being a milestone that emerges *de novo* but a pattern associated with object manipulation development, social context, and environment drivers.

### Limitations of the study

Our study provides novel insights into the developmental trajectory of tool use in children. However, we acknowledge some limitations. Firstly, our sample consisted of children from a western, or WEIRD (western, educated, industrialized, rich, and democratic^55^), population, a demographic that dominates the literature. Comparative research on small-scale societies is critical for understanding whether the developmental patterns observed here differ across cultures or reflect universal characteristics of human ontogeny (Soeters et al., in prep.). Secondly, our study design was cross-sectional and not longitudinal. While we were able to draw important age-related inferences about tool-use development, our cross-sectional design prevents causal conclusions about developmental trajectories at the individual level. Future longitudinal studies should therefore track individual tool use development over time, which would allow for direct testing of the effect of early object manipulation on later tool use. Additionally, our sample was unevenly distributed across age. Future studies with more balanced ages would allow for a more robust analysis of potential non-linear developmental trajectories. Third, individual-level variation in motor developmental milestones, such as the onset of crawling or walking, which could increase access to others and objects, was not explicitly controlled for, though age and individual identity as a random effect likely absorbed some of this variance. Lastly, a novel aspect of our study was the measurement of social and environmental drivers of tool-use development. Our coding captured opportunities for learning rather than confirmed instances of social learning, and we did not distinguish between opportunities to learn from caregivers compared to peers. Future work is needed to investigate both how children acquire tool-use skills in terms of social learning mechanisms (e.g., high vs. low fidelity^37^) as well as the models they learn from (e.g., caregiver vs. peer).

## Resource availability

### Lead contact

Further information and requests for resources should be directed to and will be fulfilled by the lead contact, Ellen Soeters (ellen.soeters@iea.uzh.ch; ellensoeters@live.com).

### Materials availability

This study did not generate new unique reagents.

### Data and code availability

• Data reported in this paper will be shared by the lead contact upon request.
• Code reported in this paper will be shared by the lead contact upon request.
• Any additional information required to reanalyze the data reported in this paper is available from the lead contact upon request.

## Acknowledgments

We thank the 10.13039/100000001Swiss National Science Foundation (PCEFP3_186967) for funding this research and the Research Ethics Committee at the University of Zurich for granting ethical approval. We are grateful to Sophie Bekins for piloting our study protocol and to Justyn Funkhouser, Siana Senn, and David Rolinger for assistance with video analyses. Special thanks go to the parents of the participating children for their trust and consent; to the caregivers who work tirelessly; and to the daycare managers for their time, collaboration, and invaluable guidance throughout this study.

## Author contributions

Conceptualization and methodology, E.S., J.A.F., C.S., and K.K.; data collection, E.S.; data curation, E.S. and J.A.F.; formal analysis, E.S., J.A.F., and E.P.W.; visualization, E.S. and J.A.F.; writing – original draft, E.S.; writing – review and editing, E.S., J.A.F., E.P.W., C.S., and K.K.; supervision, K.K.; funding acquisition, C.S. and K.K.

## Declaration of interests

The authors declare no competing interest.

## STAR★Methods

### Key resources table

**Table:** 

REAGENT or RESOURCE	SOURCE	IDENTIFIER
**Software and algorithms**		

INTERACT coding software	Mangold^56^	https://www.mangold-international.com/en
R Statistical Software (v4.2.2)	R Core Team^57^	https://www.r-project.org/
glmmTMB (package)	Brooks et al.^58^	https://CRAN.R-project.org/package=glmmTMB
ordinal (package)	Christensen^59^	https://CRAN.R-project.org/package=ordinal
DHARMa (package)	Hartig^60^	https://CRAN.R-project.org/package=DHARMa
performance (package)	Lüdecke et al.^61^	https://CRAN.R-project.org/package=performance

### Experimental model and study participant details

We recruited 31 children (17 females, 14 males) from two daycares in Zurich, Switzerland. Participants had an age range between 6 months and 58 months, with a mean age of 33 months at the beginning of the study (see Figure S1; Table S4 for age distribution details). At both daycares, children were in mixed-age groups (*n* = 5) on separate floors, each with a maximum of 10–12 children and 2–4 dedicated carers per group. Three daycare groups spanned infancy to preschool age, whilst a fourth group cared for children primarily less than 2 years of age and a fifth group children over 2 years of age; not all children in each group were recruited as participants. Children typically carried out their indoor activities within groups, but groups could mix for varying amounts of time when outdoors.

#### Ethics

Our study protocol was approved by the Research Ethics Committee at the University of Zurich (approval number: 22.12.13). Participants were recruited from two local daycares, and written informed consent was obtained from parents or legal guardians prior to data collection. Informed consent covered the child’s participation, the recording of video data for behavioral analysis, and the use of media for scientific contributions. To ensure participant confidentiality, all data were deidentified and assigned alphanumeric pseudonyms. Due to ethical restrictions relating to participant confidentiality, our datasets cannot be made publicly available. Data and corresponding scripts are available from the corresponding author upon reasonable request, subject to ethical approval.

### Method details

#### Data collection

Observational behavioral data were collected using focal sampling with continuous recording methods.^41^^,^^62^ All video focal follows (VFFs) were collected using a high-definition video camera (Panasonic VX870K). Data were collected in morning sessions (09:00–12:00) or afternoon sessions (14:00–17:00) over a 4-month period between April and July 2023 and an additional 2-month period between June and July 2024, with both data collection periods combined for analyses. A total of 27 children were observed in 2023 and 11 children in 2024, 4 of whom were new participants and 7 of whom had also been observed in 2023. Group composition remained stable within each data collection period as no children aged out or joined mid-collection. Overall, data collection accounted for a total of 68 observation days. An additional 7 days were spent at the start of data collection ensuring that participants were acclimated to the researcher’s presence and equipment.

Within each observation session, the order of VFFs was randomized by participant and we avoided recording consecutive VFFs for the same focal individual. We scored a total of 467 VFFs and each participant had a mean of 13 VFFs per data collection period (range = 5–17 VFFs; SD = 3) and 118 observation minutes (range = 44–167 min; SD = 29), totaling 74.6 h of observation time.

VFFs were recorded inside or outside the daycares. When *indoors*, each individual group had access to two playrooms, a connecting corridor, a bathroom, and a dining room. Activities were filmed either sitting or standing as still and quietly as possible to prevent disturbing the children and caregivers. The caregivers were not given specific instructions other than to act as though the researcher were not present. When *outdoors*, VFFs were recorded either in the daycares’ gardens, or in the surrounding neighborhoods (e.g., the park, the forest).

The behavioral contexts of VFFs were grouped into either free play or non-free play. The most frequent behavioral context observed was free play, in which children were able to play with toys and other children within their groups (when indoors) and between groups (when outdoors). *Free play* typically began once other activities were finished and ended when the children were encouraged by caregivers to participate in tidying up their playrooms to prepare for other activities, such as going outdoors, eating, and sleeping. Due to the limitations of data collection times, more structured behavioral contexts were recorded but were less common and thus grouped into *non-free play*. These contexts included mealtime (i.e., structured meals and informal snack time), instructional activities (e.g., story time, singing circles, arts and crafts), and traveling time (i.e., traveling through the neighborhood). Activities that were not recorded included nap time and when children were in the changing room preparing to go indoors or outdoors.

#### Video analyses

Video analyses were conducted using INTERACT coding software.^56^ With INTERACT, all variables were scored using a standardized ethogram.

##### Material engagement bouts

A *material engagement bout* was defined as when a participant actively engaged with any detached manipulatable object(s) and continuously manipulated the object with their hands, feet, or mouth^11^ for a minimum of 3 s. Bouts involving attached objects were excluded from the analyses unless the bout related to tool use (e.g., use of taps to access water, using an attached digger to dig sand). For *object manipulation* bouts, we coded the end of a bout when there was no physical contact for at least 3 s, with the exception of engagement specific to game play (e.g., kicking a football), in which a 30 s rule was applied to account for the time that children continued their object-focused play but were not in contact with the object. For all *tool use* bouts, a 30 s rule applied to account for the time when children were gathering, manufacturing, and modifying their tools before, during, and in-between their tool use bouts. Furthermore, consistent with the non-human primate literature,^20^^,^^63^^,^^64^ our coding scheme included self-directed tool use, i.e., instances where an object is used to alter the condition of the focal’s own body or deliver a substance to the self (e.g., drinking from a bottle, using a tissue to wipe a fluid; Table S5). We did not perform a sensitivity analysis excluding these early self-directed observations.

##### Material engagement scale

For each material engagement bout, we scored its *complexity level*, which is defined by the number of objects involved in a manipulation.^19^ This hierarchical scoring system increased in complexity: (1) *manipulating a single object*, (2) *manipulating multiple objects*, (3) *using a single tool*, and (4) *using multiple tools* (see Figure 1B; see definitions in Table S5). Although our scale draws on cognitive and comparative theory,^19^^,^^20^ we do not attempt to precisely proxy the cognitive demands of individual bouts. This scale provides a standardized, reproducible measure of material engagement complexity that is comparable across species and studies.

##### Social and environmental opportunities

Participants’ close proximity to others and available objects were recorded at 1-min intervals using instantaneous point sampling. Close proximity was defined as a 1 m (m) radius to the focal. At each interval, we scored the presence or absence of the following: (a) at least one individual engaged in any activity (*social proximity*); (b) at least one individual specifically engaged in object manipulation or tool use (*social opportunity*). We note that the term social opportunity captures *potential* opportunities to learn about objects or tools from others but does not measure the actual occurrence of social learning itself. For object availability: the (c) presence of *source and substrate material* (e.g., sand, attached branches); (d) presence of *natural items* (e.g., sticks, leaves); and (e) presence of *pre-manufactured toys and tools* (i.e., human-made objects). Objects being manipulated by the focal or another individual within the 1 m radius were not considered available.

At the focal-follow level (i.e., frequency model), proportions were calculated based on the number of intervals with presence divided by the total number of intervals in a VFF. At the bout level (i.e., duration and complexity models), each continuous bout was assigned the presence or absence score of the sample point that preceded the bout’s start time. This approach assured that data points reflected, as best as possible, the social and environmental context available to the participant at the beginning of the material engagement bout.

##### Interobserver reliability (IOR)

Interobserver reliability was assessed between the two video coders for manipulation bouts (K = 0.91), manipulation durations (K = 0.95), and manipulation complexity (K = 0.90). All indicators of reliability exceeded a score of 0.80.

### Quantification and statistical analysis

Our statistical analyses were completed using R (version 4.2.2^60^). We used three separate mixed-effects models to examine the frequency, duration and complexity of material engagement, each of which served as a response variable in three different models. All model predictors and response variables were specified *a priori*. No multiple testing correction was applied across our models as each model captured a distinct aspect of material engagement. Random slopes were explored during model development but led to model convergence issues, suggesting our data do not contain sufficient information to reliably estimate random slopes. We thus opted for models with random intercepts only. In addition, daycare group was initially included as a random effect but was removed from our final models as it accounted for negligible variance, suggesting that between-group differences did not meaningfully influence our results. The conditional R^2^ values we report (see Tables S1–S3) are typically modest, reflecting inherent within- and between-individual variability in naturalistic behavior, alongside potential unmeasured variance, such as weather conditions (outdoors), group size, and developmental milestones. Although our analyses are based on 447 focal follows, these observations were repeatedly sampled from 31 children. Participant ID accounts for this non-independence, but statistical power is primarily determined by variation across the 31 children, and so effect size estimates should be interpreted with appropriate caution.

We fit generalized linear mixed models (GLMMs) using the *glmmTMB* package^58^ for analyses focused on the frequency and duration of material engagement. We fit a cumulative link mixed model (CLMM) using the *ordinal* package^59^ for analysis of the complexity of material engagement. Model fit and residual diagnostics were assessed using the *DHARMa* package,^60^ and multicollinearity between predictors was assessed using the *performance* package^61^; all predictors, excluding interactions to eliminate false positives due to non-essential collinearity, returned Variance Inflation Factor values <5 indicating no problematic collinearity. Lastly, we compared each of our models to a null model using likelihood ratio tests.

In all models, *age* in months was modeled as a linear fixed effect. We constructed identical models with splines to account for potential developmental non-linearity; however, we maintained a linear fit given that the spline did not improve model parsimony (ΔAIC <7) in any of our three final models.

For our first model, we fitted a negative binomial GLMM where our continuous dependent variable was the *number of material engagement bouts* per VFF (*n* = 3223; mean = 7.21, SD = 5.48). Our fixed effects included, for social opportunities, *proportion of social proximity intervals* (mean = 0.73, SD = 0.28) and *proportion of social learning opportunity intervals* (mean = 0.54, SD = 0.35), and for object availability, proportion of *source and substrate material* (mean = 0.32, SD = 0.36)*, natural items* (mean = 0.50, SD = 0.43)*,* and *pre-manufactured toys and tools* (mean = 0.64, SD = 0.37). *Age* (mean = 33, SD = 13), *sex* (males = 14, females = 17), *location* (indoors = 126, outdoors = 321), and *behavioral context* (free play = 383, non-free play = 64) were also included as fixed effects. We included the *total duration* of each VFF as an offset (mean = 542 s, SD = 108 s) and *participant ID* (*n* = 31) as a random effect. 447 VFFs were retained for this model; 20 VFFs were omitted during modeling due to no material engagement being observed in these VFFs.

For our second model, we fitted a Gaussian GLMM with log-transformed *duration of material engagement bouts* as the continuous dependent variable (*n* = 2163; mean = 39.48 s, SD = 58.54 s). Our fixed effects included *social proximity* as a factor (*absence* = 731, *presence of social proximity* = 263, *presence of social learning opportunities* = 1169), presence of *source and substrate material* (1 = 754, 0 = 1409)*,* presence of *natural items* (1 = 1200, 0 = 963)*,* and presence of *pre-manufactured toys and tools* (1 = 1426, 0 = 737), a*ge* (mean = 35, SD = 13), *sex* (males = 1009, females = 1154), *location* (indoors = 555, outdoors = 1608), and *behavioral context* (free play = 2061, non-free play = 102). We included an interaction effect for the *type of material engagement* (object manipulation = 1596, tool use = 567) with age, with *participant ID* (*n* = 31) and *VFF ID* (*n* = 379) as random effects.

For our final model, we fitted a CLMM with *complexity of material engagement bouts* as the ordinal dependent variable (*n* = 2163; 1 = 1155, 2 = 441, 3 = 471, 4 = 96). Our fixed effects included *social proximity* as a factor (*absence* = 731, *presence of social proximity* = 263, *presence of social learning opportunities* = 1169), presence of *source and substrate material* (1 = 754, 0 = 1409)*,* presence of *natural items* (1 = 1200, 0 = 963)*,* and presence of *pre-manufactured toys and tools* (1 = 1426, 0 = 737), a*ge* (mean = 35, SD = 13), *sex* (males = 1009, females = 1154), *location* (indoors = 555, outdoors = 1608), *behavioral context* (free play = 2061, non-free play = 102), and the log-transformed *duration of material engagement bouts* (mean = −0.008, SD = 1.168). *Participant ID* (*n* = 31) and *VFF ID* (*n* = 379) were included as random effects. Proportional odds assumption could not be formally tested because formal tests have not yet been implemented for mixed models. Results should therefore be interpreted with this in mind.
