# Supplementary material for: Object manipulation scaffolds the development of tool use in children
Source: iScience. 2026 Aug 24;29(9):117302. doi: 10.1016/j.isci.2026.117302 (PMC13542482; doi:10.1016/j.isci.2026.117302)
Supplement: Supplementary file 1 [file mmc1.pdf]

iScience, Volume *29*

Supplemental information

Object manipulation scaffolds

the development of tool use in children

Ellen Soeters, Jake A. Funkhouser, Erik P. Willems, Crickette Sanz, and Kathelijne Koops

SUPPLEMENTARY MATERIALS

Supplementary Table 1. Results for a negative binomial GLMM with counts of material engagement bouts fitted as the response variable, related to STAR Methods.

Factor	Effect size (IRR)	Lower 95%	Upper 95%	Std. Error	Z Value	P Value
Age	1.01	1	1.02	0.01	2.84	0.005
Sex (male)	1.04	0.84	1.29	0.11	0.33	0.74
Location (outdoors)	0.64	0.47	0.86	0.15	-2.99	0.003
Context (non-free play)	0.4	0.32	0.49	0.11	-8.43	< 0.001
Prop. social proximity	0.57	0.39	0.83	0.19	-2.92	0.004
Prop. social learning	1.59	1.16	2.18	0.16	2.85	0.004
Prop. source/substrate	1.15	0.91	1.45	0.12	1.13	0.26
Prop. natural	1.75	1.35	2.25	0.13	4.31	< 0.001
Prop. tools	1.62	1.28	2.07	0.12	3.99	< 0.001

N_{obs} = 447, N_{ind} = 31; R^2_c = 0.054; X^2 = 128.64, DF = 12, $P < 0.001$; IRR = Incidence rate ratio coefficients are relative to the reference level or per unit increase in continuous predictors; values greater than 1 indicate increased frequency.

Supplementary Table 2. Results for a logged gaussian GLMM with durations of material engagement bouts fitted as the response variable, related to STAR Methods.

Factor	Effect size (ME)	Lower 95%	Upper 95%	Std. Error	Z Value	P Value
Age	0.99	0.99	1	0.01	-2.39	0.017
Engagement type (tool use)	0.99	0.66	1.46	0.20	-0.07	0.95
Sex (male)	1.07	0.9	1.28	0.09	0.83	0.40
Location (outdoors)	0.92	0.74	1.14	0.11	-0.79	0.43
Context (non-free play)	1.03	0.79	1.35	0.14	0.23	0.82
Presence social proximity	0.82	0.69	0.97	0.09	-2.37	0.018
Presence social learning	0.94	0.83	1.06	0.06	-1.05	0.29
Presence source/substrate	1.01	0.88	1.15	0.07	0.14	0.89
Presence natural	1	0.85	1.16	0.08	-0.04	0.97
Presence tools	0.93	0.82	1.05	0.06	-1.16	0.25
Age:Tool use	1.01	1	1.02	0.01	2.56	0.010

N_{obs} = 2163, N_{ind} = 31; R^2_c = 0.147; X^2 = 79.56, DF = 15, P < 0.001; ME = multiplicative duration effects are relative to the reference level or per unit increase in continuous predictors; values greater than 1 indicate longer durations.

Supplementary Table 3. Results for a CLMM with complexity of material engagement bouts fitted as the response variable, related to STAR Methods.

Factor	Effect size (OR)	Lower 95%	Upper 95%	Std. Error	Z Value	P Value
Age	1.02	1	1.03	0.01	2.00	0.045
Duration (log)	1.34	1.23	1.46	0.04	6.92	< 0.001
Sex (male)	0.7	0.44	1.11	0.24	-1.52	0.13
Location (outdoors)	1.84	1.15	2.96	0.24	2.52	0.012
Context (non-free play)	1.2	0.66	2.19	0.31	0.60	0.55
Presence social proximity	0.85	0.61	1.19	0.17	-0.95	0.34
Presence social learning	0.93	0.74	1.17	0.12	-0.61	0.54
Presence source/substrate	1.33	1.02	1.73	0.14	2.08	0.038
Presence natural	0.94	0.68	1.29	0.16	-0.41	0.68
Presence tools	1.44	1.12	1.85	0.13	2.82	0.005

$N_{obs} = 2163$, $N_{ind} = 31$; $R^2_c = 0.306$; $X^2 = 76.313$, DF = 10, $P < 0.001$; OR = Proportional odds ratios, relative to the reference level or per unit increase in continuous predictors; values greater than 1 indicate increased odds of higher complexity engagement.

Supplementary Table 4. Distribution of video focal follows and material engagement across age groups, related to STAR Methods.

Age group	Total children	Total VFFs	Mean VFFs per child	Mean object use bouts	SD object use bouts
0–11 months	4	55	13.75	6.14	4.38
12–23 months	5	60	12	7.44	4.26
24–35 months	13	168	12.92	6.28	4.57
36–47 months	7	81	11.57	6.81	5.36
48–59 months	9	103	11.44	8.72	5.93

Age group categories were assigned based on each child's mean age within each sampling period (2023 & 2024). Seven children contributed data in both sampling periods and therefore appear in more than one age category.

Supplementary Table 5. Definitions applied for each level of complexity in our scale of material engagement, related to STAR Methods and Figure 1.

Level	Complexity	Definition	Observed examples
1	Single item<br>*Object manipulation*	Manipulating only one single item (e.g., one rock, grass blade, toy car). Includes source material, substrate items, and items that are not uniquely discernible from one another (e.g., grass blades, pile of leaves, sand); includes bringing the manipulatable item into contact with non-detachable or manipulatable environmental features (i.e., ground, tree trunk, wall, table) or source-substrate (detached or detachable items that could be feasibly detachable by the focal – i.e., gravel, sand, branches).	Chewing on a toy, inspecting a leaf, throwing a ball
2	Multiple item<br>*Object manipulation*	Manipulating two or more items. Includes holding one object while manipulating another object, stacking multiple objects, picking up multiple objects, and simultaneously mouthing two objects (e.g., three sticks, stick and a leaf, bucket and toy cars, three blocks).	Carrying two spades, stacking Lego bricks, holding a stick and playing with a toy car
3	Single item<br>*Tool use*	Employing a single detached or manipulatable attached environmental object to alter more efficiently the form, position, or condition of another object, organism, self, source or substrate material. Includes seemingly unsuccessful attempts of tool use (Shumaker et al., 2011).	Riding a tricycle, sieving sand, wiping fluid off body with a tissue
4	Multiple item<br>*Tool use*	Employing more than one tool (as defined above).	Accessing water from a tap and filling a watering can, digging sand with a spade into a bucket, eating with a knife and fork

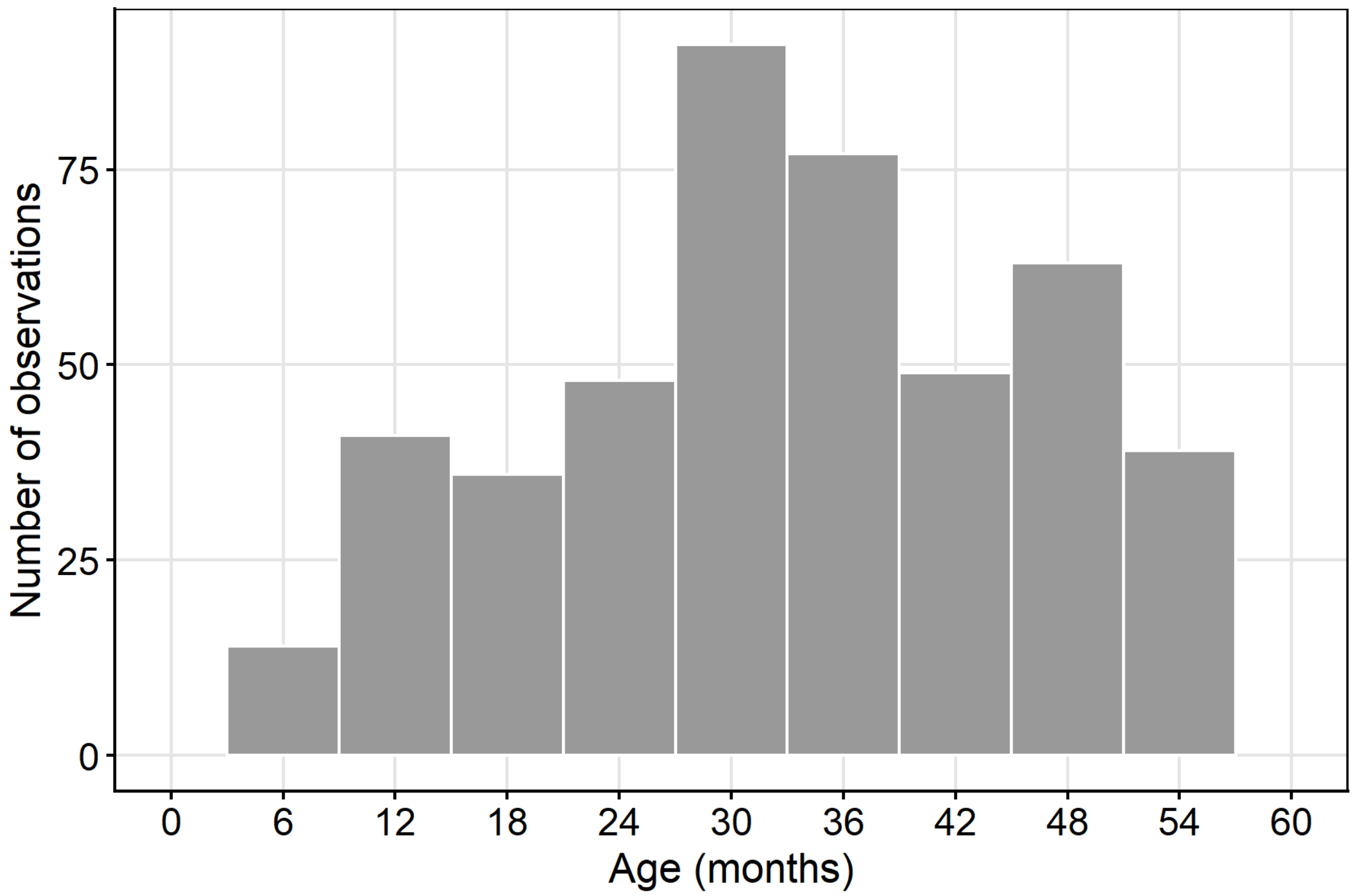

Supplementary Figure 1. Age distribution of video focal follows across the sample. Each bar represents the number of video focal follows (*n* = 467) collected within each 6-month age bin, spanning the full range of participants (6–58 months), related to STAR Methods.
